# Highly (001)-textured p-type WSe_2_ Thin Films as Efficient Large-Area Photocathodes for Solar Hydrogen Evolution

**DOI:** 10.1038/s41598-017-16283-8

**Published:** 2017-11-22

**Authors:** Farabi Bozheyev, Karsten Harbauer, Clark Zahn, Dennis Friedrich, Klaus Ellmer

**Affiliations:** 1 National Laboratory Astana, 53 Kabanbay Batyr St., 010000 Astana, Kazakhstan; 2 Institute of High Technology Physics, National Research Tomsk Polytechnic University, 30 Lenin Ave., 634050 Tomsk, Russia; 3 Helmholtz-Zentrum Berlin für Materialien und Energie, Institute for Solar Fuels, Hahn-Meitner-Platz 1, 14109 Berlin, Germany; 4 National Nanolaboratory, Al-Farabi Kazakh National University, 050000 Almaty, Kazakhstan

## Abstract

Highly (001)-textured, photoactive WSe_2_ thin films have been prepared by an amorphous solid-liquid-crystalline solid process promoted by palladium. By increasing the thickness of the Pd promoter film (≥10 nm) the structure and texture of the WSe_2_ films can be improved significantly. However, these as-crystallized WSe_2_ films are only weakly photoactive in a 0.5 М H_2_SO_4_ electrolyte under AM 1.5 solar irradiation which we attribute to an inefficient photogenerated charge transfer across the WSe_2_/electrolyte interface via the prevailing van der Waals planes of the WSe_2_ crystallites. In this work photochemically deposited platinum on the p-type WSe_2_ photocathode is used for an efficient electron transfer thus inducing the hydrogen evolution reaction. Upon illuminating the WSe_2_ photocathodes in a Pt-ion containing electrolyte, the photogenerated electrons reduce Pt^+^ to Pt leading to the precipitation of Pt islands, preferentially at edge steps of the WSe_2_, i.e. at the grain boundaries of the WSe_2_ crystallites. The increasing amount of Pt islands at the grain boundaries linearly enhances the photocurrent density up to 2.5 mA cm^−2^ at 0 V_RHE_ in sulfuric acid, the highest reported value up to now for WSe_2_ thin films.

## Introduction

Photoelectrolysis has attracted considerable attention as an alternative method to the well-known electrolysis to generate solar hydrogen in order to solve the storage problem for solar energy during dark calms^1–5^. An inherent advantage of photoelectrolysis is that it avoids the use of fossil fuels and thus the emission of carbon dioxide which is the main cause of the climate change observed since many decades.

Among the photocathode materials for the hydrogen-evolution reaction (HER), tungsten diselenide (WSe_2_) is a very promising semiconductor due to its band gap energy (indirect: E_g_ = 1.4 eV; direct: E_g_ = 1.7 eV^6^), well-matched to the solar spectrum^7–9^ and its high stability in acidic or alkaline solutions^10^. WSe_2_ exhibits a layered hexagonal structure^11^, in common with other transition metal dichalcogenides (TMDC), in which a tungsten monolayer is sandwiched between two chalcogen layers forming a triple layer of Se-W-Se by strong covalent bonding^12^. These triple layers are stacked over each other along the (001) axis, bonded by weak van der Waals forces.

In former investigations a high photoactivity of WSe_2_ was only found when single crystalline samples were used^13^. Prasad and Srivastava demonstrated that photoetching of n-WSe_2_ reduces surface defects and improves photoactive properties leading to an energy conversion efficiency in polyhalide-based redox systems of more than 17%^14^. Recently, McKone *et al*. showed that a p-type, niobium-doped WSe_2_ single crystal, coated with a Pt/Ru catalyst, splits water with a solar-to-hydrogen conversion efficiency of more than 7%^15^. They found that bare WSe_2_ crystals without a catalyst showed only a very small photocurrent, i.e. a low conversion of light to H_2_. A thin catalytically active Pt/Ru metal film on the WSe_2_ crystal surface significantly promoted the charge (electron) transfer from the cathode into the electrolyte, leading to a photocurrent density of up to 25 mA cm^−2^ at 0 V_RHE_ in an acidic solution.

In the past, it was difficult to prepare photoactive WSe_2_ films on a conducting substrate which is needed for a functioning photocathode^16–18^. Furthermore, for the large-scale application of photoelectrochemical water splitting thin film photoelectrodes are required, i.e. methods have to be developed which allow a cost-effective preparation of semiconductors on large areas^19^.

Yu *et al*. demonstrated hydrogen evolution using solution-processed WSe_2_ thin films coated with a Pt catalyst^18^. They achieved a maximum photocurrent density of about 1 mA cm^−2^. But their film deposition method is complex and leads to randomly oriented WSe_2_ flakes with large areas of thicker films, which results in a low efficiency of the photocathode. Another preparation method, first reported by Tenne *et al*., relies on a metal-promoter assisted formation of transition metal chalcogenides^16^. Here, the drawback also was, that the films could not be prepared on a conductive back contact.

Recently, we reported the two-step synthesis of p-type WSe_2_ thin films on conductive TiN:O back contacts by an amorphous solid–liquid-crystalline solid (aSLcS) process^20^. This method allows the preparation of highly (001)-textured thin films over a large area for a prospective large-scale solar energy conversion. We have shown that the (metal M) promoter MSe_x_ enhances the crystallization of amorphous WSe_2+x_ films considerably and improves the (001) texture and photoactivity of the WSe_2_ films^21–25^.

A higher photoelectrochemical cell efficiency can be achieved by improving the morphological properties, i.e. by elimination of cracks and short circuits in the film as well as of structural defects in the WSe_2_ crystallites that lead to recombination of photoexcited electron-hole pairs. Another challenge of these semiconductors with van der Waals surfaces is the charge transfer to water molecules adsorbed at the surface as shown recently by McKone *et al*.^15^. For MoS_2_, Jaramillo *et al*. have shown that the most active sites for electrochemical H_2_ evolution are the edges of MoS_2_ nanoparticles, where the electrocatalytic activity correlated linearly with the number of edge sites on the hexagonal MoS_2_ platelets^26^. This points to the effect of specific sites (at the non-van-der-Waals planes) that mediate an efficient charge transfer at the semiconductor/electrolyte interface.

In the present work we demonstrate the highly textured crystallization of WSe_2_ films on a conductive back contact and that the photodeposition of Pt islands improves the photoactive properties of polycrystalline WSe_2_ thin films significantly, allowing the application of these films for solar hydrogen generation. Additionally, the Pt deposition mechanisms on WSe_2_ and its influence on the solar-to-hydrogen evolution are discussed.

## Results and Discussion

### Structural and morphological properties

**Figure 1:**
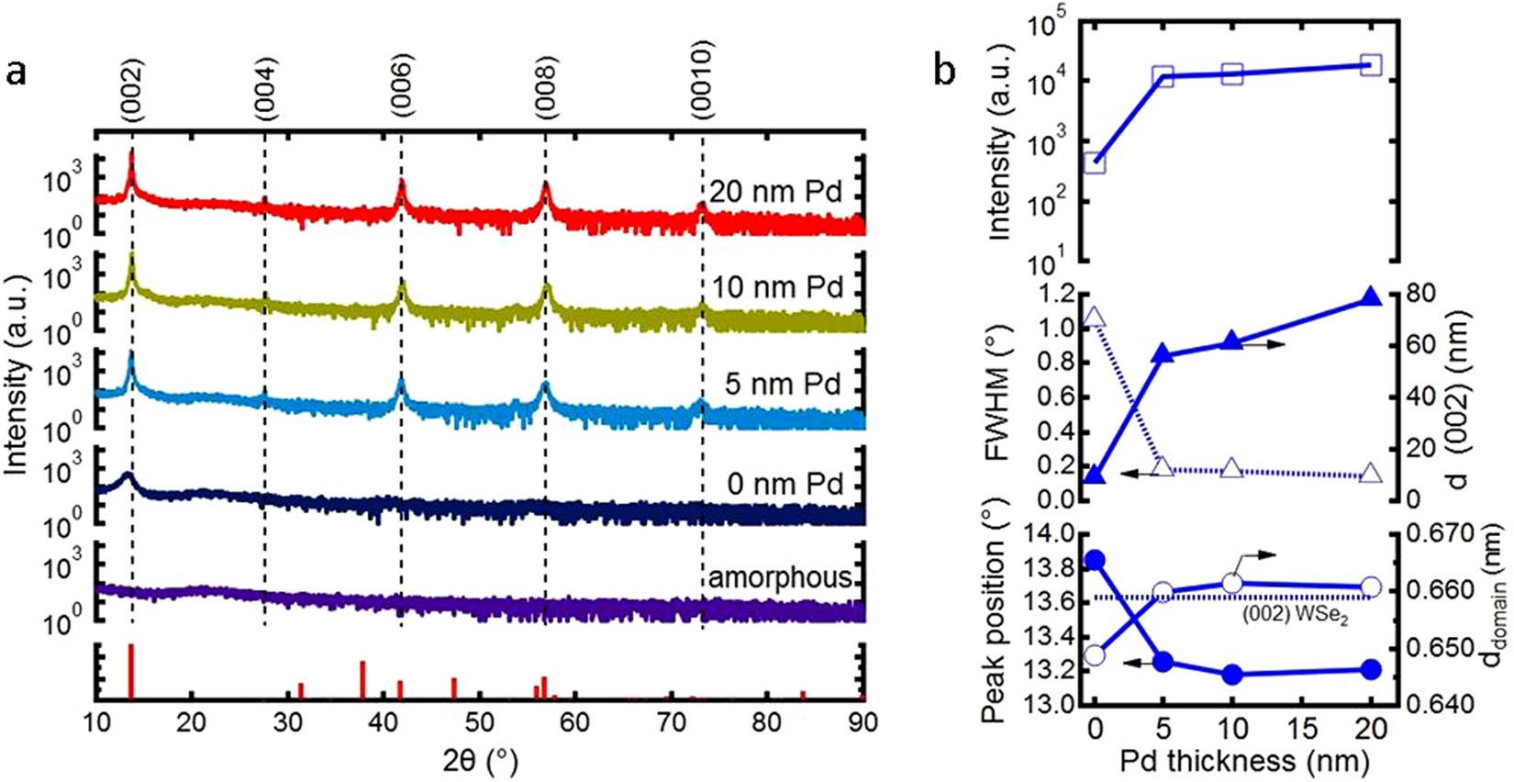
Structure of the tungsten selenide films. (**a**) X-ray diffraction patterns of WSe_2_ films, crystallized with Pd-promotion on quartz glass at different Pd-promoter thicknesses (0 to 20 nm). The Pd-assisted crystallized films exhibit only (002 l) (l = 1–5) diffraction peaks, showing the strong (001)-texture of the films. The bar diagram at the bottom displays the powder diffraction pattern of WSe_2_ (JCDPS no. 38–1388). For comparison the XRD pattern of a Se-rich film deposited at room temperature is also shown which, obviously, is X-ray amorphous. (**b**) Pd thickness dependence of the (002)-peak intensity, full width at half maximum (FWHM), and the peak position. The WSe_2_ film thicknesses are 120 ± 10 nm except of the X-ray amorphous film which had a thickness of about 340 nm. The samples were crystallized at 550 °C at a pressure of 1.25 Pa for Ar:H_2_Se (partial pressure ratio 1:4).

**Figure 2:**
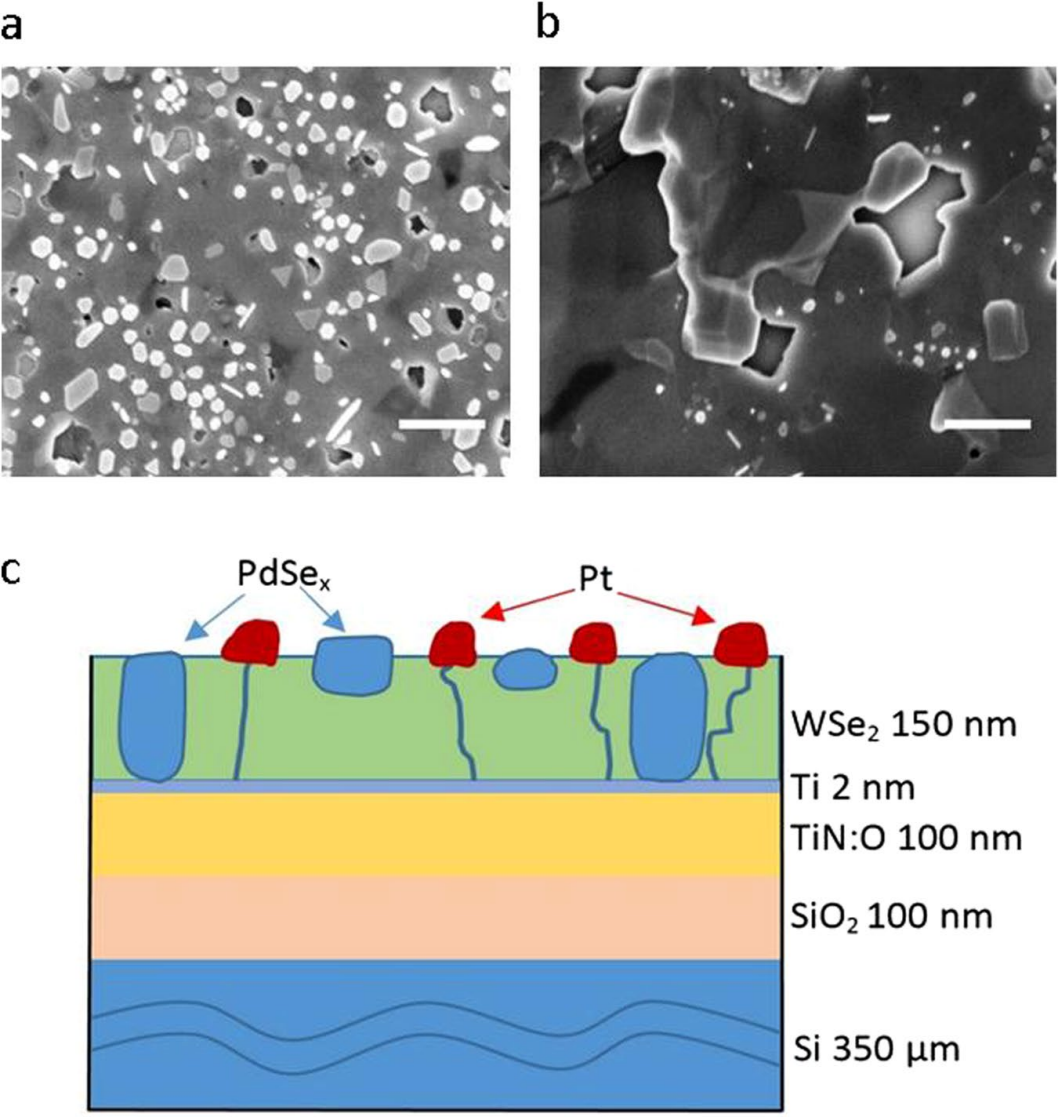
Surface morphology of the WSe_2_ films. SEM images of WSe_2_:Pd films in top view on TiN:O/SiO_2_/Si substrates (scale bar is 500 nm). The films were crystallized with (**a**) 10 nm and (**b**) 20 nm Pd-promoter thicknesses, respectively. The samples were crystallized at 550 °C at a gas pressure of 1.25 Pa for Ar:H_2_Se (partial pressure ratio 1:4). The WSe_2_ film thicknesses are 120 ± 10 nm. (**c**) Schematic cross section of the photoelectrode configuration: Pt-WSe_2_:PdSe_x_/Ti/TiN:O/SiO_2_/Si. The Pt nanoislands are preferentially photodeposited at the grain boundaries of WSe_2_.

The structural properties of WSe_2_ films grown by the aSLcS process were reported already in our recent paper^20^. In the following only the dependence of the structural film parameters on the Pd-promoter thickness is shown, see Fig. 1. With increasing thickness of the Pd-promoter film the crystalline quality of the WSe_2_ films is considerably improved, which is obvious from the increase of the (002 *l*)-peak (*l* = 1–5) intensities by several orders of magnitude, and the corresponding decrease of the full widths at half maximum (FWHM) of the (002) diffraction peaks. Both parameters saturate for promoter thicknesses above 10 nm. The intensity and FWHM of the (002) diffraction peaks of Pd-promoted films almost approach the values for standard LaB_6_ powder (NIST, SRM 660B) emphasizing a perfect texturized structure of the films (see Fig. S1). The FWHM is only twice as large as the instrumental width of the diffractometer pointing to the large coherently diffracting domains in (001) direction. The average grain sizes, i.e. the size of the coherently diffracting domains, are larger for films, promoted at the highest promoter thicknesses. The (002)-peak positions of the WSe_2_ films asymptotically approaches the peak position for a standard powder diffraction pattern of WSe_2_ (JCPDS no. 38–1388), which means that the lattice parameters of the WSe_2_ film are close to the parameters of a WSe_2_ single crystal. The structure of the WSe_2_ films is slightly Se-deficient, since the position of the (002)-peak is shifted slightly to larger 2θ values with respect to the positions of the standard powder WSe_2_ diffraction pattern, which was also observed for the WSe_2_ films in our previous work^20^. As the Pd thickness is increased from 10 nm up to 20 nm the lateral size of the crystallites is enlarged, demonstrating the promotion effect of the Pd, see Fig. 2a,b. The average lateral size for the best crystallized WSe_2_ films is about 1–2 µm, inferred from the SEM pictures. Regula *et al*. studied the peak width of the (002) diffraction peak of a Ni promoted WS_2_ film crystallized at 850 °C as a function of the Ni thickness^21^. Here, the FWHM decreased from 0.8° down to 0.18°, as the thickness of Ni was increased from 0 to 10 nm. We observe a similar decrease in FWHM from 1° to 0.14° as the thickness of Pd is increased from 0 to 20 nm. The schematic cross section of the WSe_2_ films, crystallized with Pd-promoter on Ti/TiN:O/SiO_2_/Si substrates is depicted in Fig. 2c.

**Figure 3:**
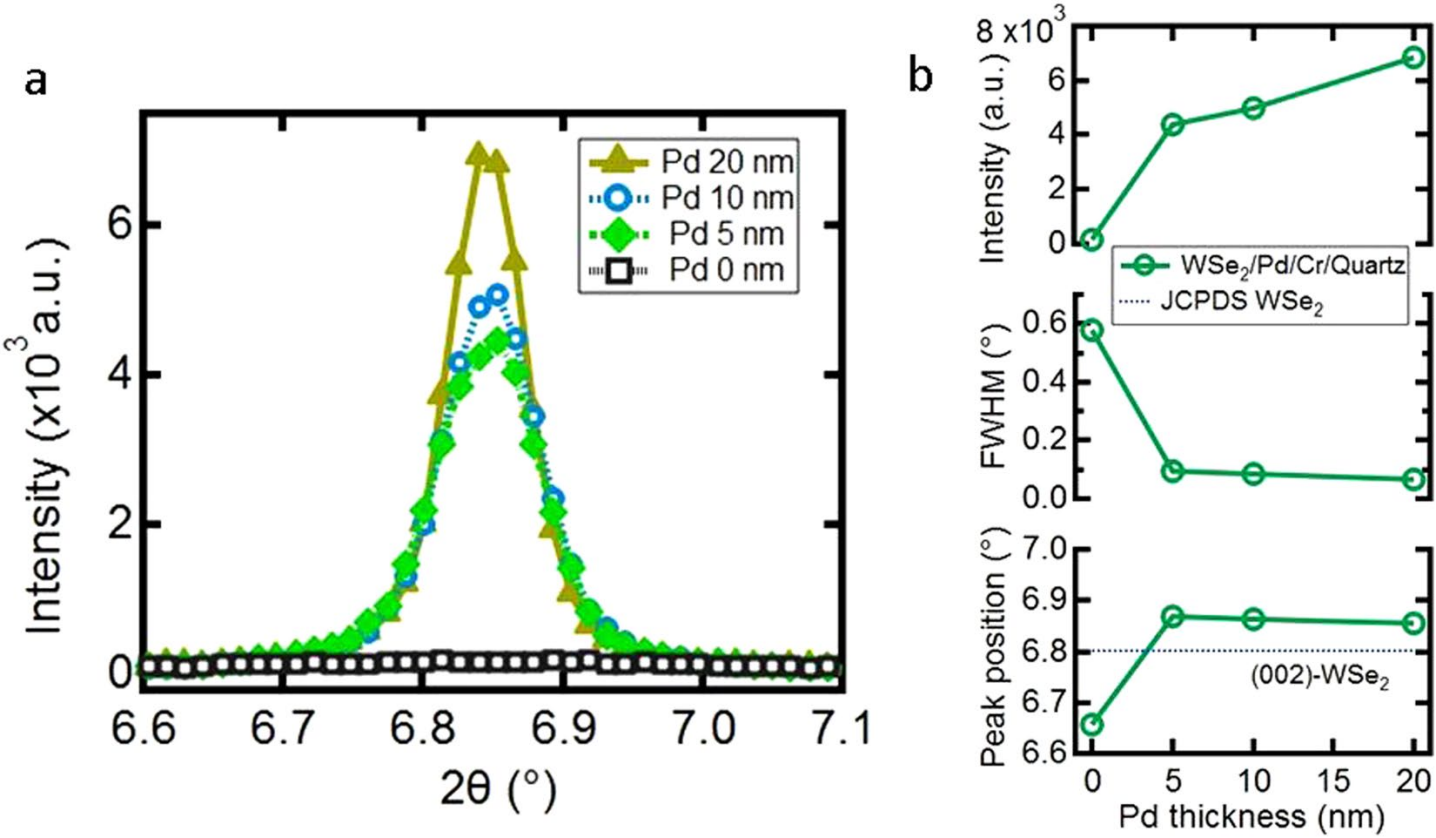
Rocking curves of the WSe2 films. (**a**) Distribution of domain orientation of WSe_2_ films, crystallized with Pd-promotion on quartz glass at different Pd-promoter thicknesses. (**b**) The characteristic parameters of the rocking curves - (002)-peak intensity, full width at half maximum, and peak position – as a function of the Pd thickness. The WSe_2_ film thicknesses are 120 ± 10 nm.

The degree of the (001) texture of the crystallites was investigated by rocking curve measurements of the (002) reflection at θ = 6.84°, see Fig. 3a. All films, crystallized with Pd-promotion, show very narrow rocking curve widths pointing to the excellent structural quality of the WSe_2_ crystallites. The FWHM of the rocking curves reaches a minimum of 0.07°, which is comparable to values typically obtained for epitaxial thin films^27^.

### Optical and photoactive properties

**Figure 4:**
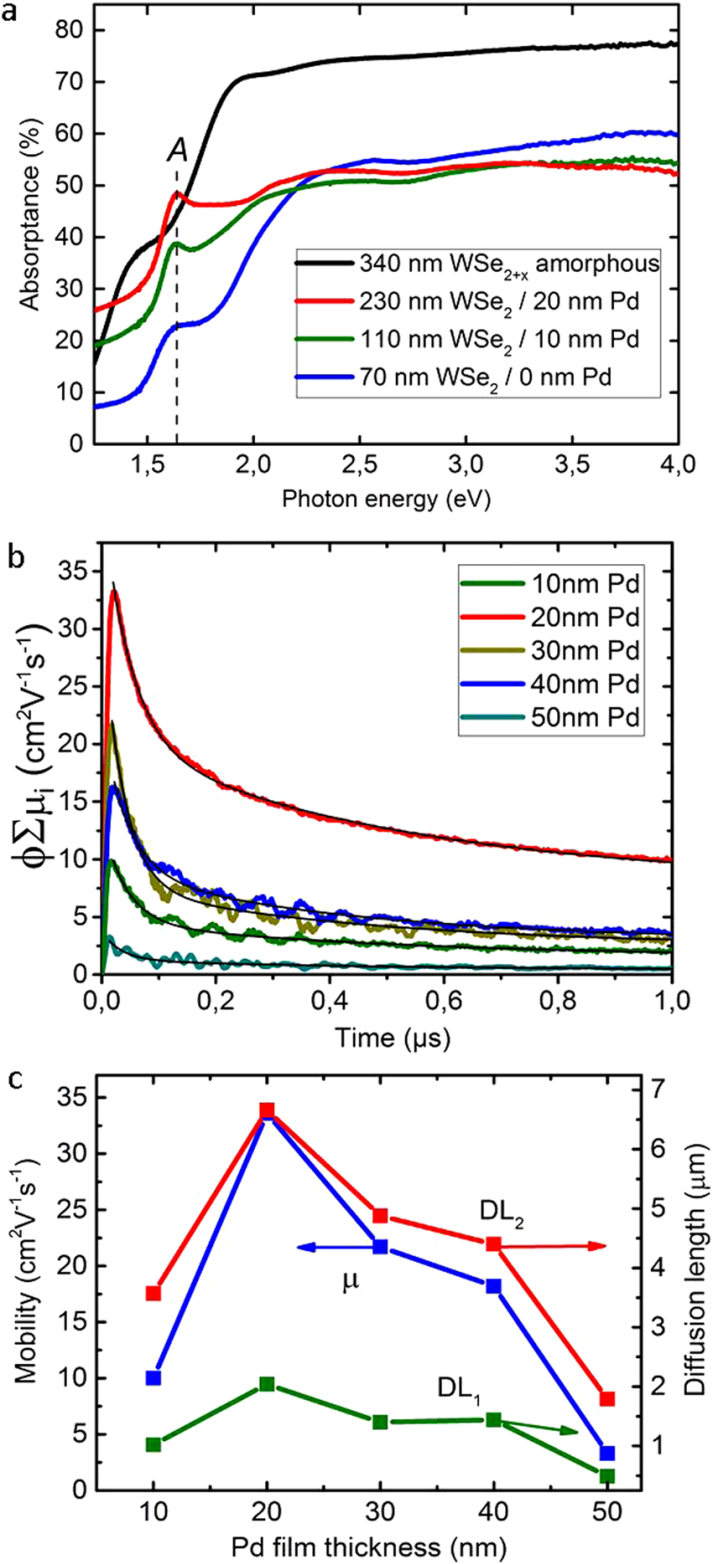
Optical and photoactive properties of tungsten selenide films. (**a**) Absorbance spectra of an amorphous Se-rich WSe_2+x_ film and of WSe_2_ films, crystallized without and with 10 and 20 nm Pd-promoter layers at a temperature of 550 °C and a pressure of 1.25 Pa for Ar:H_2_Se (partial pressure ratio 1:4). (**b**) Time resolved microwave conductivity signals of WSe_2_ films (200 ± 50 nm thick), crystallized with 10, 20, 30, 40 and 50 nm thick Pd-promoter at 550 °C and 10 Pa of H_2_Se, respectively, and (**c**) their TRMC parameters: the sum of the mobilities (ϕΣµ_i_)_max_ and diffusion lengths DL_1_ and DL_2_ of electron-holes (excitation yield ϕ~1) as a function of the Pd thickness.

The optical absorption properties of the tungsten selenide films were studied in the photon energy range of 1.2–4 eV (Fig. 4a). After annealing at 550 °C, the thickness of an amorphous 340 nm WSe_2+x_ film is dramatically changed to 70 nm (5 times) caused by the evaporation of the excess Se. This was proved by Rutherford backscattering spectroscopy studies in our previous paper^20^, where the Se-to-W ratio changed significantly from about 10 to 2 (i.e. WSe_2_) after annealing above 300 °C. As can be seen in Fig. 4a the absorbance of the film is reduced with decreasing WSe_2_ thickness. The crystallization of amorphous WSe_2+x_ films with 10 and 20 nm Pd layers, respectively, leads to larger thicknesses than that of the films crystallized without Pd-promoter due to the formation of larger WSe_2_ domains (see Fig. 1b) and PdSe_x_ crystallites. Therefore, the absorption of the thicker WSe_2_ films crystallized with the 20 nm of Pd layer tends to have larger absorbance values in the photon energy range of 1.2 to 2.3 eV. At higher Pd layer thickness (20 nm) a sharpening of the *A* exciton peak at 1.63 eV is observed, which is attributed to the direct transitions from the split valence band to the conduction band^12^, demonstrating the improved electronic structure of the film. The maximum measured absorption coefficient of the films is in the order of 10^5^ cm^−1^ (Fig. S2). This extraordinarily high absorption coefficient of the films is caused by the characteristic bandgaps for the TMDC like MoS(e)_2_ and WS(e)_2_^6^ in the range of 1.5–1.9 eV.

The increase of the Pd promoter thickness (>20 nm) leads to an improvement of the crystaline structure of the WSe_2_ films (Figs S3 and S4) and to the formation of the Pd-rich metallic phase PdSe_x_, which can act as recombination centers and/or short circuits^20^. The absorbance spectra of the WSe_2_ films, crystallized with the increasing Pd thicknesses from 10 to 50 nm, do not change considerably, however the photoactivity of the WSe_2_ films varies much, which was proved by time resolved microwave conductivity (TRMC) mesurements^28^. The lifetimes of the charge carriers for the fast and slow decay components of the WSe_2_ films (50 ns and 0.5 µs) are of the same order of magnitude as for MoS_2_ and WS_2_ nanopowders, measured by the pulsed cathodoluminescence method^9,29,30^. The short lifetime is attributed to band-to-band recombination, whereas the slow component is connected with trap states in the bandgap. Increasing the Pd thickness from 20 nm to 50 nm results in a significant reduction of the mobility of the carriers from 34 to 3 cm^2^ V^−1^s^−1^ and their diffusion lengths DL_1_ from 2 to 0.5 µm (DL_2_ from 6.7 to 1.8 µm) due to the recombination of the photoexcited electron-holes at the metallic PdSe_x_ phase. Thus, the optimum Pd thickness for crystallization of WSe_2_ film can be concluded to be 20 nm, which leads to films with better structural and electronic qualities (Figs 1 and 4).

### Photoelectrochemical performance

The WSe_2_ films in the as-crystallized state, though exhibiting a highly (001)-textured morphology, showed only a marginal photocurrent (less than 0.1 mAcm^−2^ at 0 V vs. RHE). This is in accordance with a recent measurement of the photoactivity of WSe_2_ single crystals where also only a tiny photocurrent was observed for a bare WSe_2_ crystal^15^. Yu *et al*., on the other hand, observed for their nanoflake WSe_2_ films, which exhibit a large number of grain boundaries, a low photocurrent also in the as deposited state, i.e. without applying a catalyst^18^. This indicates that the surface and morphology of our WSe_2_ films is comparable to that of WSe_2_ single crystals with respect to the role of crystallographic defects, i.e. grain boundaries, for the charge transfer from the solid to the electrolyte.

**Figure 5:**
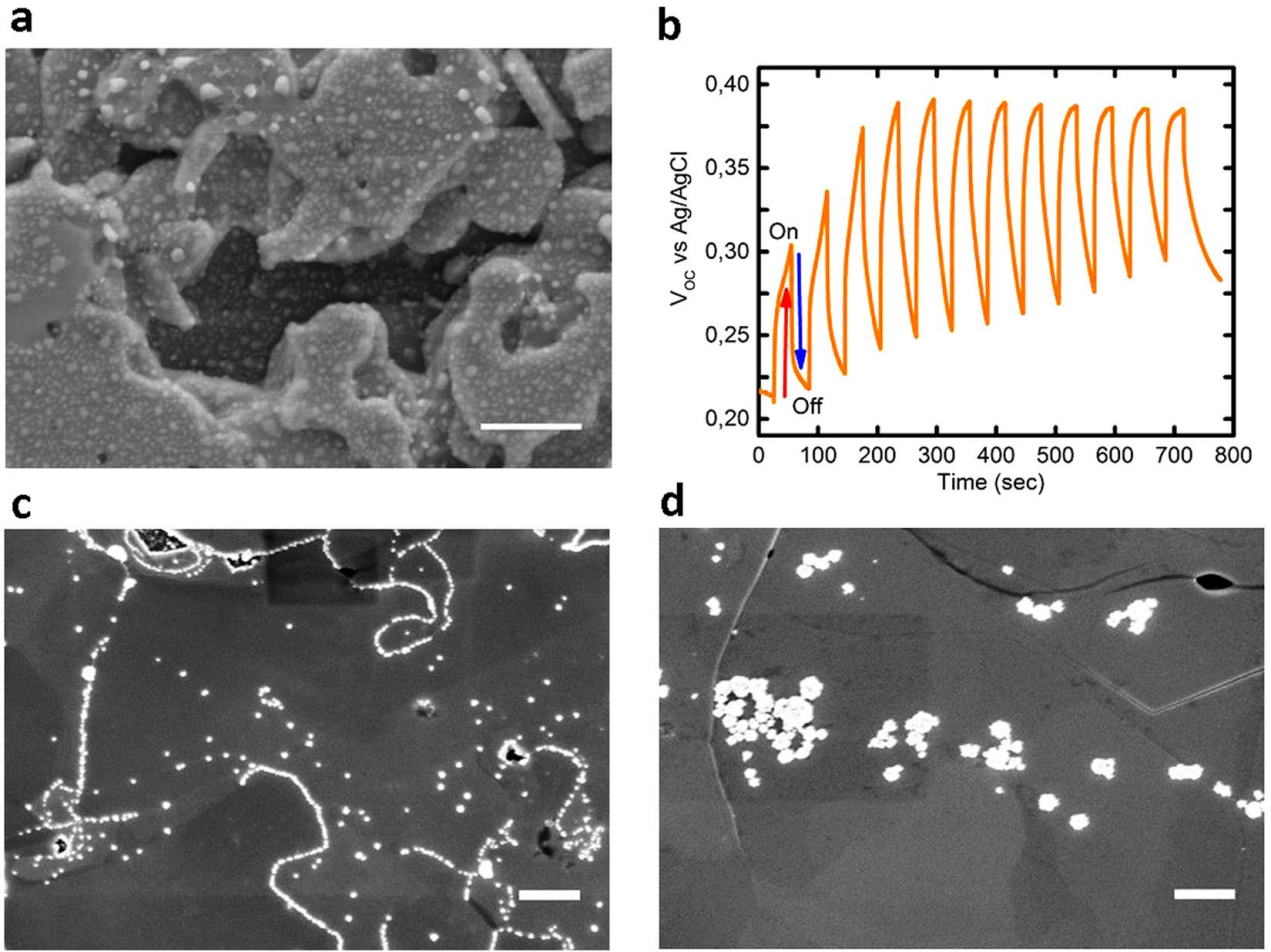
Surface morphology of Pt-photodeposited WSe2 films. (**a**) SEM image of a Pt-coated (by electron beam evaporation) etched WSe_2_ film (2 nm thick Pt layer). (**b**) The open circuit potential V_OC_ change during the periodic exposure (12 cycles) to AM 1.5 light in 0.1 M HCl. (**c**,**d**) SEM images of photochemically Pt-coated 100 nm thick WSe_2_ films at (**c**) 6 cycles (180 sec) and (**d**) 12 cycles (360 sec) in top view, respectively. All scale bars are 500 nm.

In order to increase the photocurrent of our WSe_2_ films, Pt nanoislands were precipitated on top of the WSe_2_ films, at first by electron beam evaporation at room temperature (Fig. 5a). Instead of a homogenous thin film, Pt islands were formed which are distributed quite homogenously on the WSe_2_ surface. However, we could not observe an increase of the photocurrent. On the other hand, the photodeposition of Pt islands at open circuit potential in an electrolyte (0.1 M HCl with Pt salt) led to a significant increase of the photocurrent density (Fig. 5). At light on conditions, the open circuit potential (OCP) is raised up to its maximum value due to the increasing number of photogenerated electron-hole pairs; at light off conditions the electron-holes pairs recombine, and consequently the OCP decreases (Fig. 5b). During the illumination of the WSe_2_ photocathode the Pt ions in the electrolyte react with the photogenerated electrons and are deposited at the most active sides of the WSe_2_ crystallites, i.e. at the grain boundaries (see Figs 5c and S6). At OCP conditions the electric field induced at the surface of the WSe_2_ film by electrons causes the Pt ion flow from the electrolyte to the photocathode surface (local flow), i.e. there is no current flowing in the external circuit. Due to the strong crystallographic and electrical anisotropy of the WSe_2_ crystallites, the photogenerated charge carriers are transported within the basal {001}-planes, i.e. perpendicular to the (001)-direction, eventually reaching the grain boundaries where they can be transferred to the electrolyte inducing an electrochemical reaction. Thus, the Pt islands are growing in the regions of the highest electron current, i.e. at the edges/grain boundaries of the WSe_2_ crystallites. An increase of the Pt-deposition time (number of photodeposition cycles) leads to an increase of the number and the size of the Pt islands on the WSe_2_ films (see Fig. 5c,d), which results in an increase of the photocurrent density. This is clearly visible from Fig. 5c which shows photochemically deposited Pt islands after a deposition time of 180 s, preferentially formed at edges/grain boundaries. After 360 sec photodeposition (Fig. 5d) bigger Pt-islands were grown on top of the crystallites as well as at the grain boundaries.

The morphology of the WSe_2_ films varies considerably in dependence on the crystallization pressure of the H_2_Se gas (Figs 2b and 5c). The crystallization at a lower pressure (1.25 Pa) leads to films with smaller crystallite sizes (1–2 µm) compared to that (4–5 µm) of films crystallized at higher pressures (10 Pa). This is obviously due to the compensation (suppression) of the evaporation pressure of Se from the WSe_x_ films by higher H_2_Se gas pressures resulting in the formation of larger crystallites. Etching of the films in aquaregia (for 30 sec) increases the number of edge sides and decreases the crystallite size (Fig. 5a).

**Figure 6:**
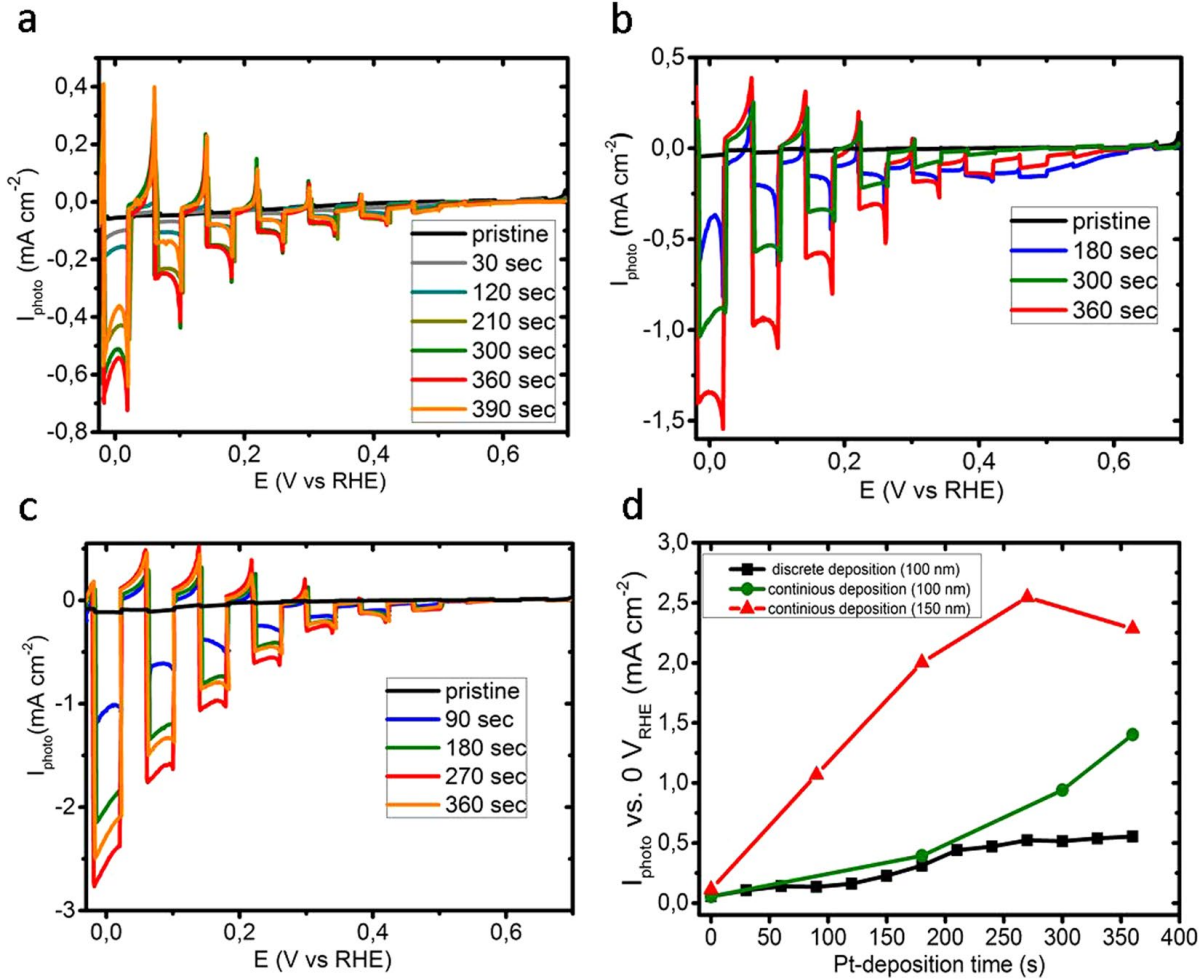
Photoelectrochemical performance of the WSe2 cathodes. Chopped (light/dark) I-V scans for 100 nm thick WSe_2_ thin films in 0.5 М H_2_SO_4_ electrolyte at (**a**) discrete and (**b**) continuous Pt-deposition procedures. (**c**) Chopped (light/dark) I-V scans for 150 nm thick WSe_2_ thin films crystallized on a 30 nm thick Pd film in 0.5 М H_2_SO_4_ electrolyte at different continuous Pt-deposition times. (**d**) Dependence of the photocurrent density at 0 V_RHE_ for different Pt-deposition times. Each light-on cycle corresponds to 30 sec light exposure.

In Fig. 6 the chopped (light/dark) I-V curves of the pure and Pt-deposited WSe_2_ films are displayed. The pure WSe_2_ film exhibited only a very small photocurrent in sulfuric acid. This can be attributed to the fact that the edge sites (i.e. the grain boundaries) of the crystallites are the sites where the photocurrent is transferred to the WSe_2_/electrolyte interface, the density of which is quite low in our large-grained WSe_2_ films. This is in agreement with a recent publication of Jaramillo *et al*.^26^ who identified the edge sites of MoS_2_ crystallites, also a layer-type semiconducting material, structurally very similar to WSe_2_, as the catalytically active sites. The increase of the photocurrent with increasing Pt deposition time shows that the Pt catalyst improves the reaction kinetics, i.e. the rate of electron transfer to the electrolyte. The fast decrease of the photocurrent (see the spikes in Fig. 6) after switching on the light, indicates a recombination of charge carriers.

Due to the small discrete islands compared with the area measured, it is difficult to exactly determine the sizes of the islands. Nevertheless, we tried to evaluate the average Pt thickness by energy dispersive X-ray fluorescence spectroscopy (EDX). The average thickness of the platinum on the WSe_2_ film, assuming a homogenous distribution over the entire surface area, equals to about 1 nm, which is in the range of the sensitivity limit of the method (Fig. S7 and Tables S2–S5). Since the surface coverage is in the order of 4% only, the size of each Pt island is in the order of 90 nm. The maximum photocurrent density is reached after 12 cycles of Pt deposition. Further increasing the Pt thickness leads to a decrease of the photocurrent, which we attribute to the increased shadowing of the WSe_2_ film by the opaque Pt islands (see below).

A continuous Pt-photodeposition under open circuit conditions leads to a better performance of the WSe_2_ films (Fig. 6d), since each I-V testing in 0.5 M H_2_SO_4_ after each Pt-deposition cycle influences the further formation of Pt nanoislands. We assume that an interruption of Pt-deposition followed by IV-testing (charge transfer through Pt nanoislands) in 0.5 M H_2_SO_4_ leads to precipitation of unfunctionalized Pt nanoislands (see Fig. 6a,b). The highest photocurrent density of 1.4 mA cm^−2^ is reached after 12 continuous cycles, which is significantly higher than the value of 0.56 mA cm^−2^ shown in Fig. 6a. An increase in the WSe_2_ film thickness from 100 nm to 150 nm resulted in a further increase in the photocurrent density from 1.4 mA cm^−2^ to 2.5 mA cm^−2^ (Fig. 6c), which is due to a better absorption of the light (Fig. 4) and consequent generation of a higher concentration of the electron-hole pairs. For this film we also observed the shadowing effect by increasing the number of Pt nanoislands at maximum deposition time (Fig. S8), which leads to a gradual decrease of the photocurrent density.

### IPCE spectrum

**Figure 7:**
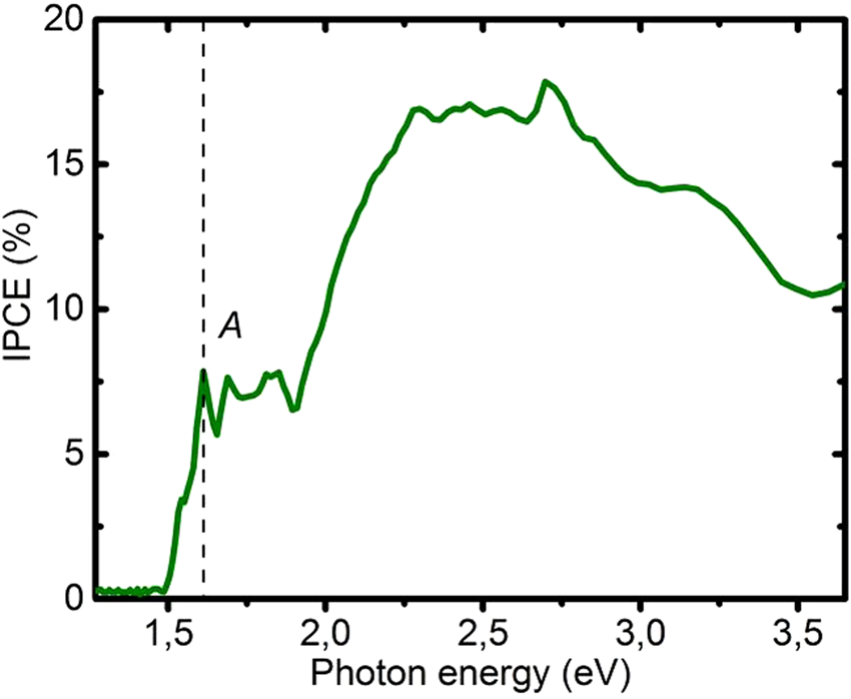
Spectral sensitivity of the WSe2 photocathode. IPCE of a WSe_2_ photocathode coated with an ATM catalyst.

The incident-photon-to-current (IPCE) spectrum for a WSe_2_ film with a catalyst (ammonium thiomolybdate-ATM^31^), comparable to platinum, is shown in Fig. 7. The conversion efficiency is low (7–17%), pointing to the still improvable intrinsic quality of our WSe_2_ films; for comparison, the WSe_2_ single crystals of McKone *et al*. exhibit an external quantum effiency of 50 to 60%^15^.

The spectral features of the IPCE curve (Fig. 7) resemble that of the absorption spectrum of WSe_2_, i.e. the observed photocurrent is due to the absorption in the WSe_2_ film. Especially remarkable is the appearance of the A exciton at about 1.6 eV^7^. This exciton has a high binding energy of about 55 meV, explaining its visibility at room temperature. The photocurrent density of the WSe_2_ photocathode, calculated from the IPCE curve is about 3.1 mA cm^−2^, which is close to the 2.5 mA cm^−2^ measured under AM1.5 illumination.

### H_2_ evolution

**Figure 8:**
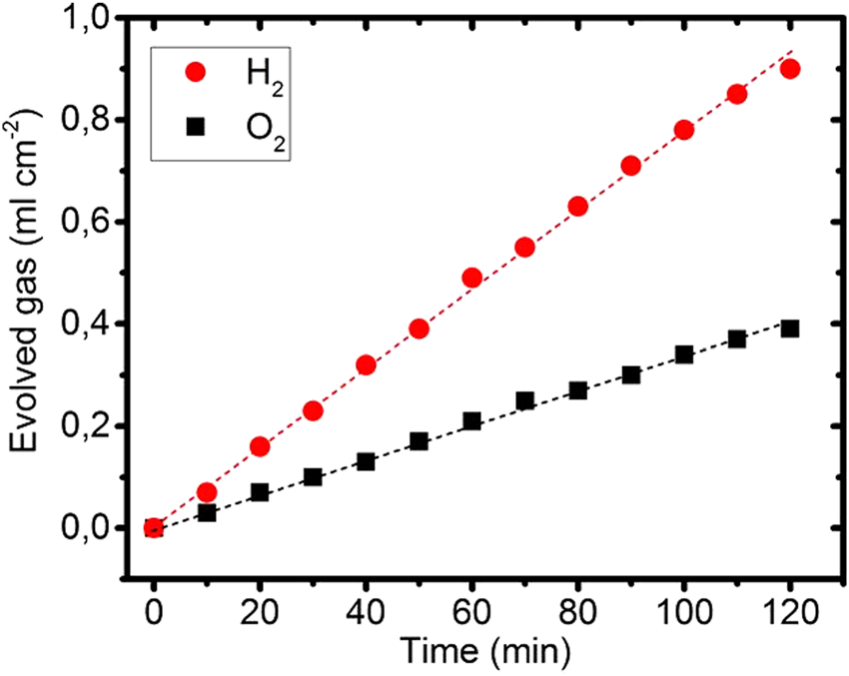
Gas production by the WSe_2_ photocathode. H_2_ and O_2_ gas evolution at a WSe_2_ photocathode coated with an ATM catalyst over a duration of 2 h.

The H_2_ production of the WSe_2_ photocathode under AM1.5 illumination was measured with a two burette system with manual pressure compensation according to King and Bard^32^, see Fig. 8. For the H_2_ evolution, 2 electrons and for O_2_ production 4 electrons are required, respectively, i.e. the volume of evolved H_2_ must be twice as much as the O_2_ volume^33^. This relation is fulfilled in our case where we obtain a volume ratio of 2.2. The WSe_2_ photocathode was operated at a lower photocurrent density of about 1.25 mA cm^−2^ due to the high serial resistance between the two burettes, which were connected with a narrow 8 cm long glass tube. The overall electrical charge transferred, was about 9 Coulomb, which had produced 1.8 ml of H_2_ gas under illumination of a 2 cm^2^ photocathode area during 2 hours, see Fig. 8. From these data a Faradaic efficiency of 81% can be calculated, which shows a high transition rate of electrons to produce hydrogen H_2_ from the H^+^ ions.

### Surface analysis

**Figure 9:**
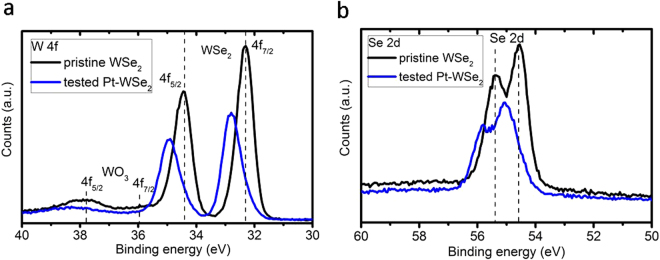
XPS of the WSe2 films. XPS spectra of (**a**) the as-prepared WSe_2_ and (**b**) Pt-coated (390 sec) WSe_2_ films tested during 13 cycles upon illumination (1.5 AM) in 0.5 М H_2_SO_4_.

The surface properties of the WSe_2_ films were tested by the X-ray photoelectron spectroscopy (XPS), see Fig. 9a,b. The XPS spectra of the WSe_2_ film show the presence of two tungsten-containing phases: the prominent peaks at 32.25 eV and 34.4 eV can be attributed to the WSe_2_, the weaker peaks at 35.7 eV and 37.85 eV are due to a small amount of tungsten oxide similar to other reports^34,35^. The doublet peaks at 54.5 eV and 55.3 eV correspond to only selenium environment present in the film. The XPS spectra of the as-prepared and the photoelectrochemically tested WSe_2_ films revealed an oxidation of the film after 13 cycles of IV-testing (Fig. 6), where both W and Se peaks are shifted to higher energies (ΔE = 0.5 eV). Azcatl *et al*.^36^ studied the surface of WSe_2_ upon ultraviolet (UV)–O_3_ exposure by *in situ* XPS, where the WSe_2_ layer was oxidized to WSe_x_O_y_. Similar to this report we observe the shifted Se 3d and W 4f peaks at 55.8 eV and 32.8 eV assigned to the WSe_x_O_y_ phase (55.4 eV and 33.1 eV), which actually proves the photocorrosion of the WSe_2_ film after IV-testing in 0.5 М H_2_SO_4_ acid upon illumination.

## Conclusions

We have improved the structural quality and the photoactivity of highly (001)-textured WSe_2_ films on quartz glass and on TiN:O back contacts, prepared by the amorphous solid-liquid-crystalline solid crystallization process, assisted by a Pd promoter layer, which was reported recently by us^20^. Increasing the thickness of the Pd film improves the structure and the photoactivity of the WSe_2_ films. However, the as-crystallized WSe_2_ films are only weakly photoactive at AM 1.5 solar irradiation in a 0.5 М H_2_SO_4_ electrolyte which we attribute to an inefficient photogenerated charge transfer to the WSe_2_/electrolyte interface via the dominating van der Waals planes of the WSe_2_ crystallites.

Only after photochemical deposition of Pt, resulting in Pt nanoislands deposited preferentially at the WSe_2_ grain boundaries, a significant photocurrent was observed that is attributed to hydrogen evolution. This result can be explained by the fact that WSe_2_ edge sites (i.e. mostly the grain boundaries) of the crystallites are the active sites where the photogenerated electrons are transferred to the WSe_2_/electrolyte interface. This is in agreement with a recent publication of Jaramillo *et al*.^26^ who identified the edge sites of MoS_2_ crystallites, also a layer-type semiconducting material, isostructural with WSe_2_, as the catalytically active sites.

An interplay between deposition parameters like the film and metal-promoter thicknesses, crystallization pressure and temperature, obviously influences on the grain sizes and their orientation, crystal structure, optical and electronic qualities of the WSe_2_ films. Our Pt-coated WSe_2_ films exhibit a photocurrent density in an acidic electrolyte (0.5 М H_2_SO_4_) up to 2.5 mA cm^−2^ under AM1.5 illumination, the highest photocurrent density reported up to now for thin WSe_2_ films. These encouraging results open a new large-area-scalable preparation route for photocathodes for solar hydrogen evolution.

## Methods

### Synthesis of the WSe_2_ films

The highly (001)-textured tungsten diselenide thin films have been prepared by the amorphous solid-liquid-crystalline solid (aSLcS) process on pure quartz glass (QG) and on oxidized Si substrates coated with a TiN:O metallic back contact. The preparation details have been reported recently; therefore only a short description is given here^20,24^. At first, X-ray amorphous, Se-rich WSe_2+x_ (x ≫ 1) films were deposited by reactive magnetron sputtering from a tungsten target in an Ar/H_2_Se atmosphere at room temperature onto a thin Pd-promoter film onto the chemically inert TiN:O back contact layer. Afterwards these amorphous films were crystallized by the aSLcS process in an H_2_Se atmosphere at a pressure of about 10 Pa at a substrate temperature of 550 °C during 10 min. In order to improve the adhesion of the WSe_2_ film a 2 nm thin Ti or Cr film was deposited by electron beam evaporation (EBE) onto the TiN:O back contact. The surface temperatures of the quartz and the TiN:O/SiO_2_/Si substrates were calibrated relative to the heater temperature (Fig. S9). A schematic cross section of the Pt-WSe_2_:PdSe_x_/TiN:O/SiO_2_/Si photocathode is shown in Fig. 2c.

### Film characterization methods

The film structure, the phase composition and the texture quality (derived from rocking curves) were characterized by X-ray diffraction (XRD) using a PANalytical XPert MPD diffractometer with CuKα radiation (0.15408 nm), while the film morphology and the element distribution were analyzed by scanning electron microscopy (SEM) and energy dispersive X-ray fluorescence analysis (EDX) with a LEO GEMINI 1530 electron microscope.

UV−vis measurements were carried out with a PerkinElmer Lambda 950 double-beam spectrophotometer in the wavelength range from 300 to 1000 nm.

Time resolved microwave conductivity (TMRC) measurements of the films were carried out by a 10 ns (FWHM) frequency-doubled Q-switched Nd:YAG laser pulse using a 532 nm wavelength and at an intensity of 1.28 × 10^11^ photons cm^−2^.

The chemical composition of the WSe_2_ film surface was studied by X-ray photoelectron spectroscopy (XPS) using a monochromatic Al K_α_ source (1486.74 eV).

### Photochemical Pt deposition

1 mM K_2_PtCl_4_ was dissolved in 0.1 M HCl solution in order to deposit Pt on top of WSe_2_/Pd/TiN:O layer stack. The photodeposition of Pt was carried out at open circuit potential (≈0.2 V) under continuous or periodic light exposure. In the latter case, the shutter was opened and closed every 30 seconds.

### Photoelectrochemical measurements

These measurements were performed in a three-electrode set-up using a potentiostat (EG&G Princeton Applied Research, model 273 A) with an Ag/AgCl (saturated KCl and AgCl solution) reference electrode and a Pt coil as counter electrode. The illumination was provided by a solar simulator (WACOM, model WXS-505-5H; AM1.5, 100 mWcm^−2^). For contacting the WSe_2_ film, a metallic wire was attached to the TiN:O back contact with an adhesive Ag metal sheet. In order to improve the contact, an In-Ga (liquid) eutectic was added between the metal sheet and the back contact TiN:O. The cyclic voltagrams were taken in the potential range from 0.5 V to −0.3 V with a scan rate of 20 mV s^−1^. Polycrystalline films of WSe_2_/Pd with thicknesses from 100 to 150 nm were the working electrode (cathode). The sample was periodically illuminated with an exposure and dark time of 2 sec each. The exposed electrode area was 0.24 cm^2^.

## Electronic supplementary material

Supporting information
