# Author Correction: Highly (001)-textured p-type WSe_2_ Thin Films as Efficient Large-Area Photocathodes for Solar Hydrogen Evolution

**DOI:** 10.1038/s41598-018-24795-0

**Published:** 2018-05-25

**Authors:** Farabi Bozheyev, Karsten Harbauer, Clark Zahn, Dennis Friedrich, Klaus Ellmer

**Affiliations:** 1 National Laboratory Astana, 53 Kabanbay Batyr St., 010000 Astana, Kazakhstan; 2 Institute of High Technology Physics, National Research Tomsk Polytechnic University, 30 Lenin Ave., 634050 Tomsk, Russia; 3 Helmholtz-Zentrum Berlin für Materialien und Energie, Institute for Solar Fuels, Hahn-Meitner-Platz 1, 14109 Berlin, Germany; 4 National Nanolaboratory, Al-Farabi Kazakh National University, 050000 Almaty, Kazakhstan

Correction to: *Scientific Reports* 10.1038/s41598-017-16283-8, published online 22 November 2017

Clark Zahn and Dennis Friedrich were omitted from the author list in the original version of this Article. This has been corrected in the PDF and HTML versions of the Article, and the Supplementary Information file that accompanies the Article.

The Acknowledgements section now reads:

“The authors would like to thank Fanxing Xi for SEM analysis and the ATM deposition, Moritz Kölbach for SEM/ EDX analysis, Paul Plate for XPS, and Sean Berglund for his help with the PEC measurements. The research association Optotransmitter-Umweltschutz-Technologie e.V. (Berlin, Germany) is acknowledged for the preparation of the TiN:O back contact layers. F.B. acknowledges the Ministry of Education and Science of the Republic of Kazakhstan for a research fellowship under the program no. 0115РК03029 “NU-Berkeley strategic initiative in warm-dense matter, advanced materials and energy sources for 2014–2018” and grants from Tomsk (Russia) Polytechnic University Competitiveness Enhancement Program and National Nanotechnology Laboratory of open type of Al-Farabi Kazakh National University, Almaty (Kazakhstan), 0263/PSF.“

The Author Contributions section now reads:

“K.E. and F.B. conceived the study. F.B. and K.H. performed the deposition experiments. F.B. did the XRD and PEC measurements of the films. C.Z. and D.F. measured and analyzed the TRMC data. F.B. and K.E. analyzed the data. F.B. drafted the manuscript and K.E. finished the manuscript using the contributions from the other authors.”

