# Supplementary material for: Highly (001)-textured p-type WSe2 Thin Films as Efficient Large-Area Photocathodes for Solar Hydrogen Evolution
Source: Sci Rep. 2017 Nov 22;7:16003. doi: 10.1038/s41598-017-16283-8 (PMC5700039; doi:10.1038/s41598-017-16283-8)
Supplement: Supplementary file 1 — Supporting information [file 41598_2017_16283_MOESM1_ESM.pdf]

Highly (001)-textured p-type WSe_2 Thin Films as Efficient Large-Area Photocathodes for Solar Hydrogen Evolution

Farabi Bozheyev[1,2,3,4], Karsten Harbauer[3], Clark Zahn[3], Dennis Friedrich[3] and Klaus Ellmer[3]

[1]National Laboratory Astana, 53 Kabanbay Batyr St., 010000 Astana, Kazakhstan

[2]Institute of High Technology Physics, National Research Tomsk Polytechnic University, 30 Lenin Ave., 634050 Tomsk, Russia

[3]Helmholtz-Zentrum Berlin für Materialien und Energie, Institute for Solar Fuels, Hahn-Meitner-Platz 1, 14109 Berlin, Germany

[4]National Nanolaboratory, Al-Farabi Kazakh National University, 050000 Almaty, Kazakhstan

Supplementary information

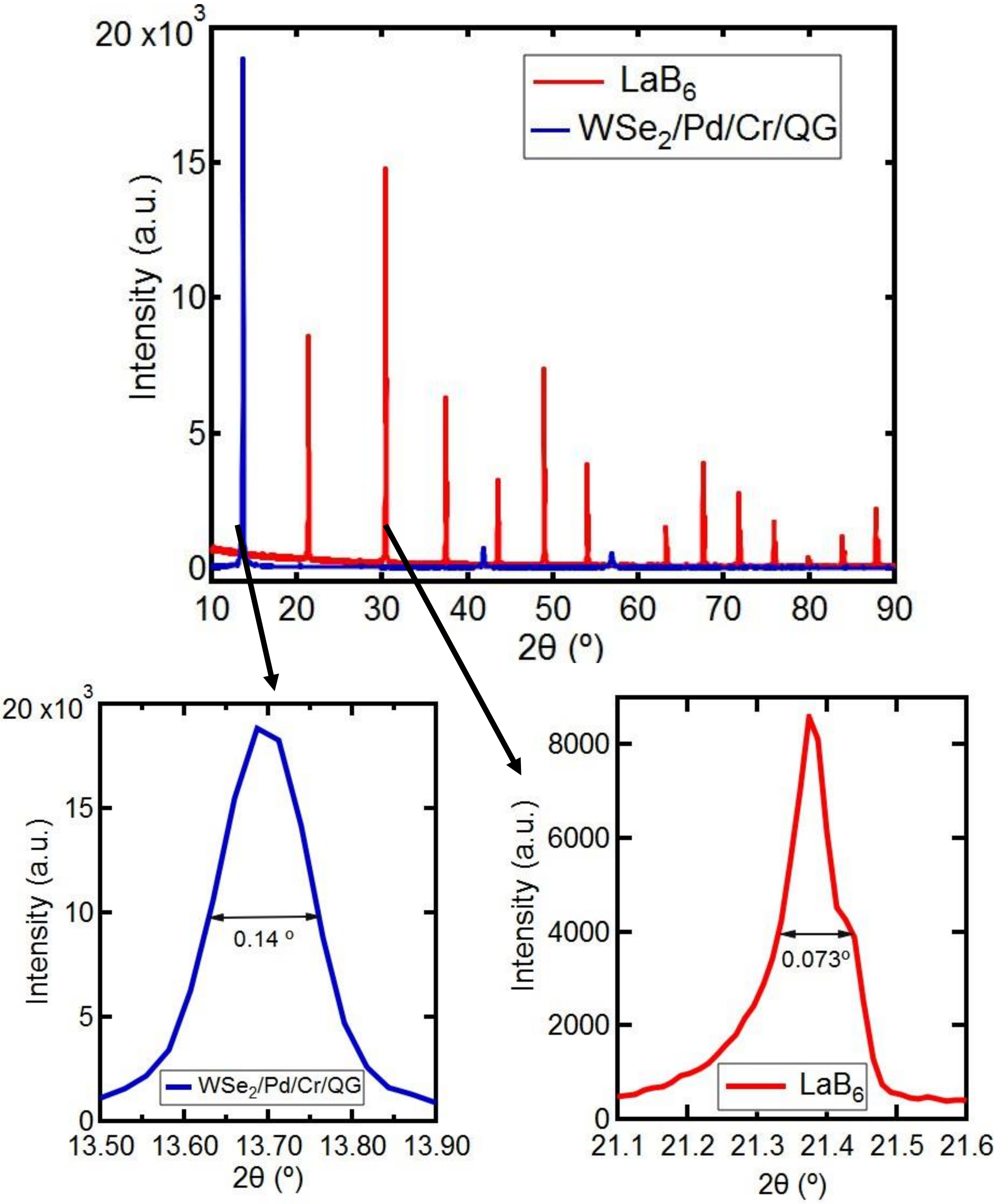

Figure S1. XRD patterns of LaB_6 single crystal and WSe_2/Pd/Cr/TiN:O film crystallized at 550 °C. Below are the (002)-peak of WSe_2 and the nearest LaB_6 peak.

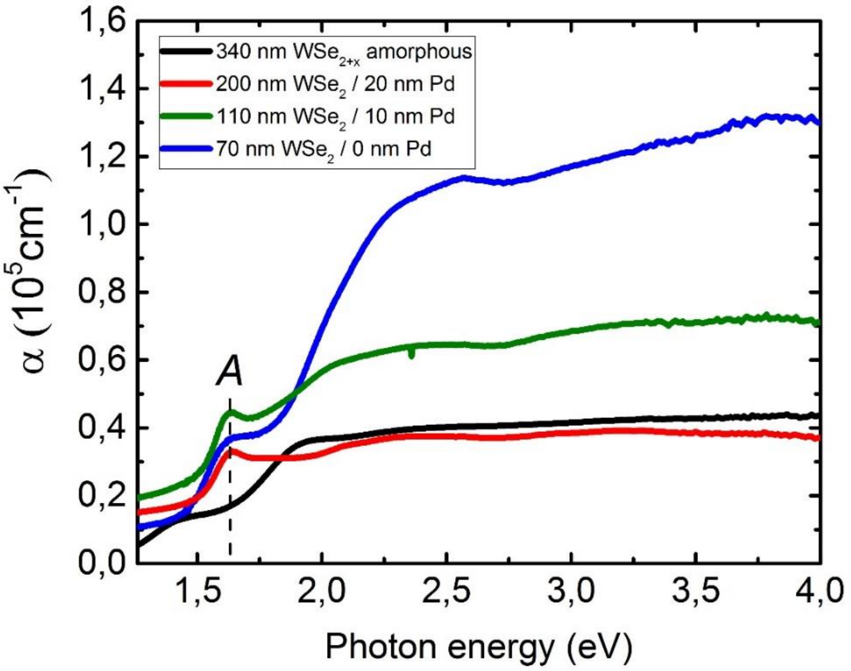

Figure S2. Absorption coefficient as a function of the photon energy for an amorphous Se-rich WSe_{2+x} film and WSe_2 films, crystallized without and with 10 and 20 nm Pd-promoter layers at 550 °C and 1.25 Pa of Ar:H_2Se (partial pressure ratio 1:4).

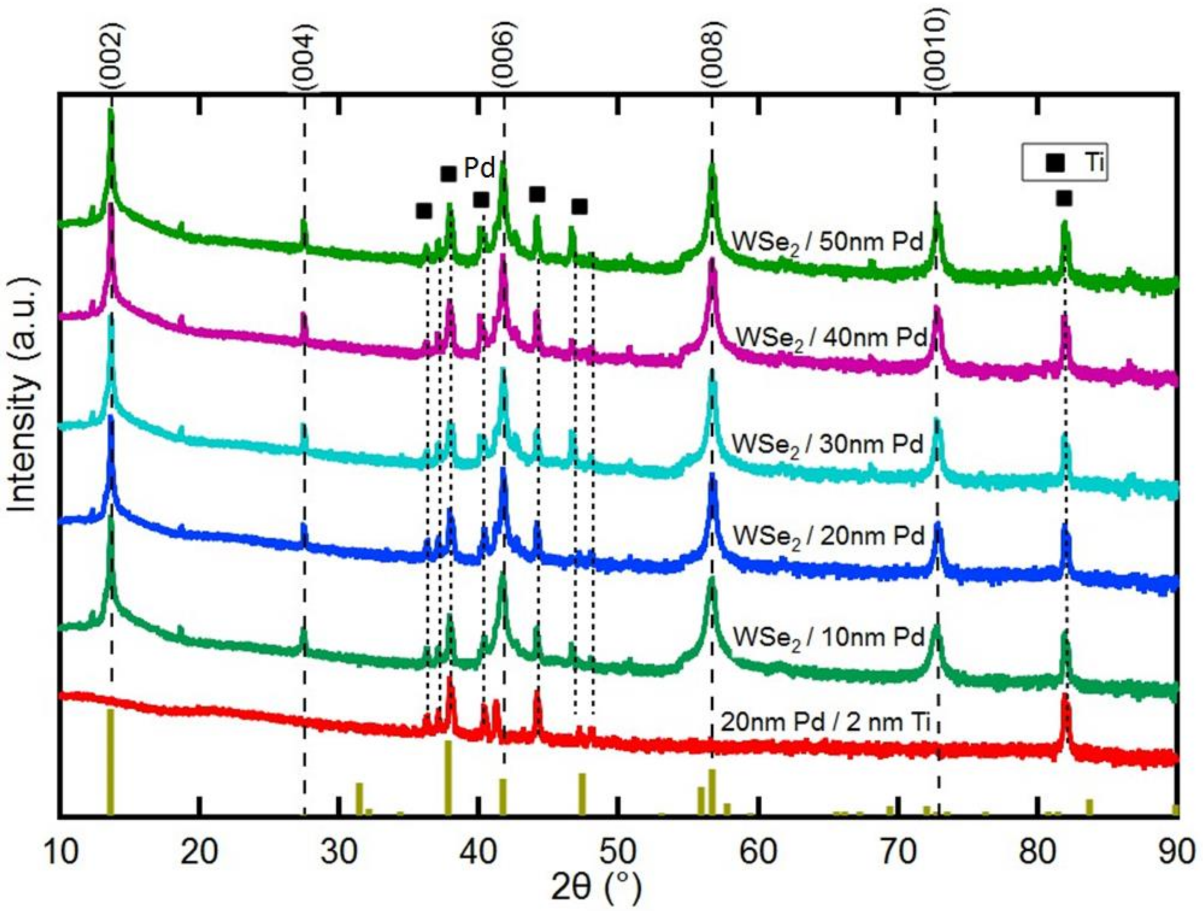

Figure S3. X-ray diffraction patterns of WSe_2 films, crystallized with Pd-promotion on quartz glass at different Pd-promoter thicknesses (10 to 50 nm). The Pd-assisted crystallized films exhibit the Pd and Ti phases, and (002l) (l = 1 – 5) diffraction peaks, showing the strong (001)-texture of the films. The bar diagram at the bottom displays the powder diffraction pattern of WSe_2 (JCDPS no. 38-1388). The WSe_2 film thicknesses are 200 ± 50 nm. The samples were crystallized at 550 °C at a pressure of 10 Pa for Ar:H_2Se (partial pressure ratio 1:4).

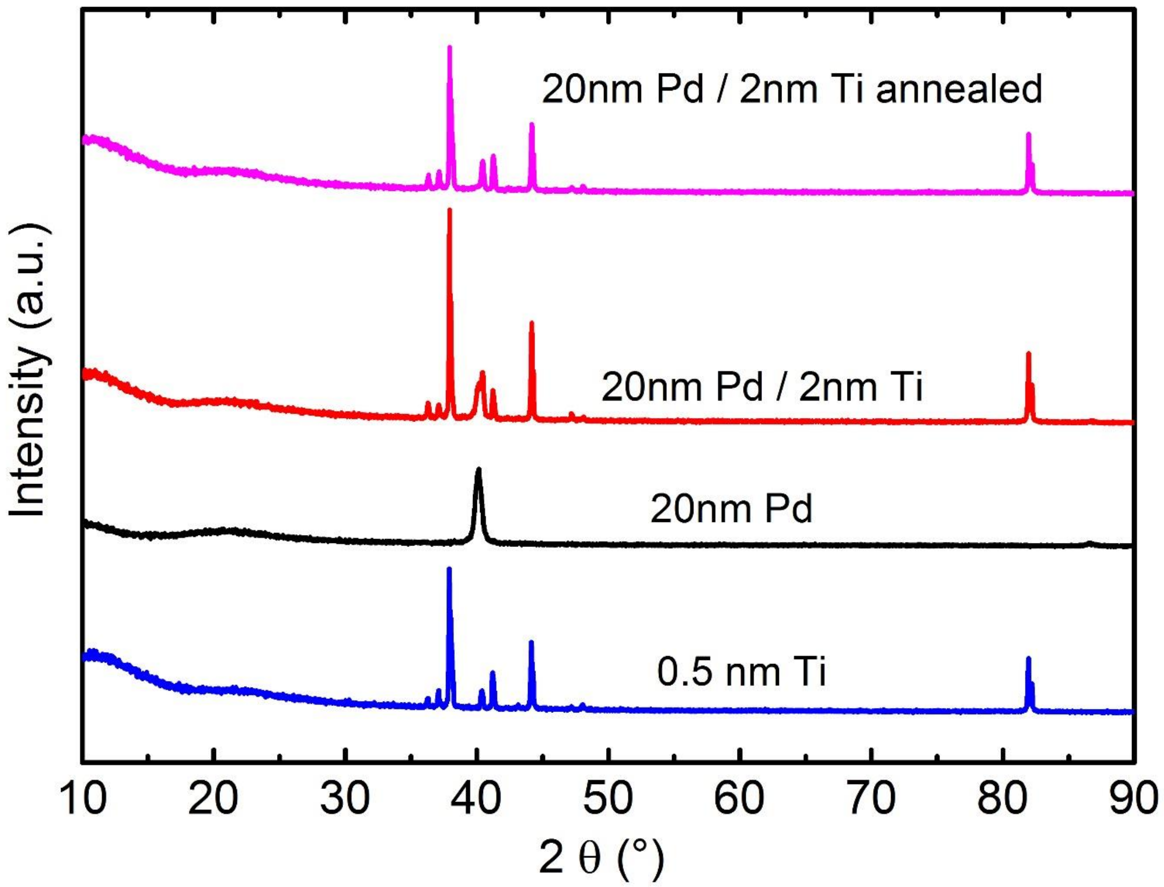

Figure S4. X-ray diffraction parrterns of 0.5 nm Ti, 20 nm Pd, 20 nm Pd / 2 nm Ti films, and, 20 nm Pd / 2 nm Ti film annealed under 10 Pa of H_2Se at 550 °C. All films were deposited by electron beam evaporation on quartz glass substrates at room temperature. Annealing of the 20 nm Pd / 2 nm Ti in 10 Pa of H_2Se at 550 °C leads to a partial evaporation of Pd and Ti resulting in a decrease in the intensity of Ti peaks.

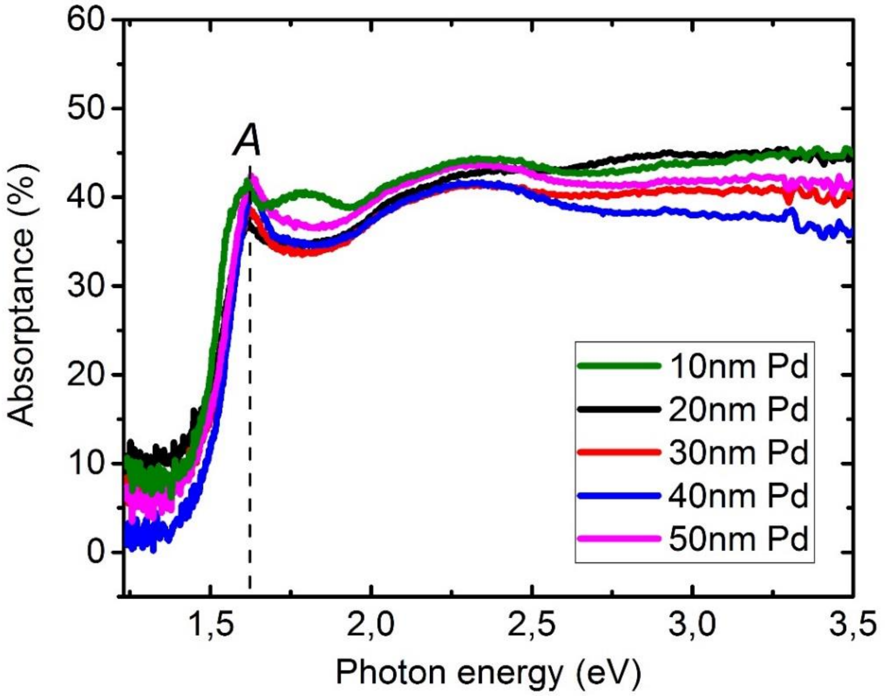

Figure S5. Absorption spectra as a function of the photon energy for 200 ± 50 nm thick WSe_2 films crystallized with Pd-promoter of different thickness from 10 to 50 nm at 550 °C and 10 Pa of H_2Se.

Table S1. TRMC parameters: mobility, lifetimes and diffusion lengths of electron-holes as a function of the Pd thickness.

WSe_2 sample crystallized	Mobility	Lifetimes		Diffusion lengths	
	μ (cm^2 $V^{-1}s^{-1}$)	τ_1 (ns)	τ_2 (ns)	L_{D1}	L_{D2}
10 nm Pd	10	42	511	1.02	3.57
20 nm Pd	33.6	49	522	2.04	6.66
30 nm Pd	21.7	37	450	1.4	4.88
40 nm Pd	18.2	45	421	1.44	4.40
50 nm Pd	3.3	34	448	0.49	1.79

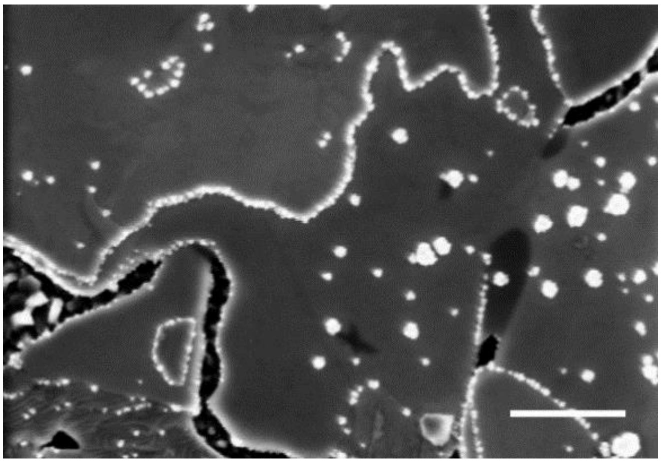

Figure S6. SEM images of a Pt-coated WSe_2 film by photochemical precipitation (180 sec). Scale bar is 500 nm.

Supplementary Tables

Table S2. EBE deposited 2 nm thick Pt on WSe_2/Pd/Ti/TiN:O

Element	Scan area 1, at. %	Scan area 2, at.%	Scan area 3, at. %	Average scan, at. %	Avarege error, at.%
O K	4.91	6.41	9.35	6.89	0.25
Ti K	2.98	2.53	3.19	2.9	0.19
Se L	57.91	57.64	55.19	56.91	0.26
W M	32.83	32.56	31.42	32.27	0.22
Pt M	1.37	0.86	0.86	1.03	0.15

Table S3. EBE deposited 5 nm thick Pt on WSe_2/Pd/Ti/TiN:O

Element	Scan area 1, at. %	Scan area 2, at.%	Scan area 3, at. %	Average scan, at. %	Average error, at.%
N K	12.53	13.25	14.71	13.50	0.79
O K	10.1	9.86	11.22	10.39	0.4
Ti K	24.19	23.18	22.09	23.15	0.27
Se L	17.66	16.33	14.14	16.04	0.14
Pd L	4.85	7.55	8.84	7.08	0.18

W M	26.61	25.91	25.32	25.95	0.17
Pt M	4.07	3.92	3.68	3.89	0.12

Table S4. EBE deposited 10 nm thick Pt on WSe_2/Pd/Ti/TiN:O

Element	Scan area 1, at. %	Scan area 2, at.%	Scan area 3, at. %	Average scan, at. %	Average error, at.%
N K	3.68	3.05	3.34	3.36	0.73
O K	8.91	9.82	9.09	9.27	0.37
Ti K	5.74	5.37	5.45	5.52	0.2
Se L	44.51	44.44	44.5	44.48	0.22
Pd L	3.55	3.81	5.05	4.14	0.14
W M	24.11	23.96	23.59	23.89	0.19
Pt M	9.51	9.56	8.98	9.35	0.16

Table S5. Photodeposited 1 nm thick Pt (12 cycles) on WSe_2/Pd/Ti/TiN:O

Element	Scan area 1, at. %	Scan area 2, at.%	Scan area 3, at. %	Average scan, at. %	Average error, at.%
N K	20.67	19.4	20.6	20.22	0.66
O K	11.97	11.58	11.5	11.68	0.37
Ti K	28.07	30.06	28.13	28.75	0.22
Se L	9.38	11.97	9.56	10.30	0.10
Pd L	6.78	2.68	6.91	5.46	0.09
W M	22.68	23.82	22.9	23.13	0.12
Pt M	0.45	0.49	0.4	0.45	0.04

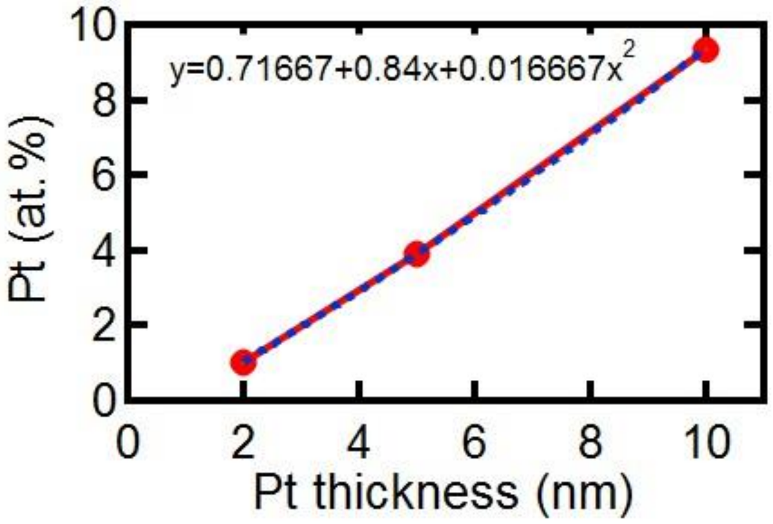

Figure S7. Evaluation of the photodeposited Pt thickness. EDX analysis of the surfaces of the WSe_2 films coated by Pt of 2, 5 and 10 nm thicknesses, respectively, using electron beam evaporation, where the thicknesses of the films were measured by quartz crystal monitor. From this calibration curve, the average thicknesses of the photodeposited Pt-layer have been evaluated to be 1 nm (0.5 at.%) for 12 cycles, respectively. The scan area corresponds to 100x100 μm^2.

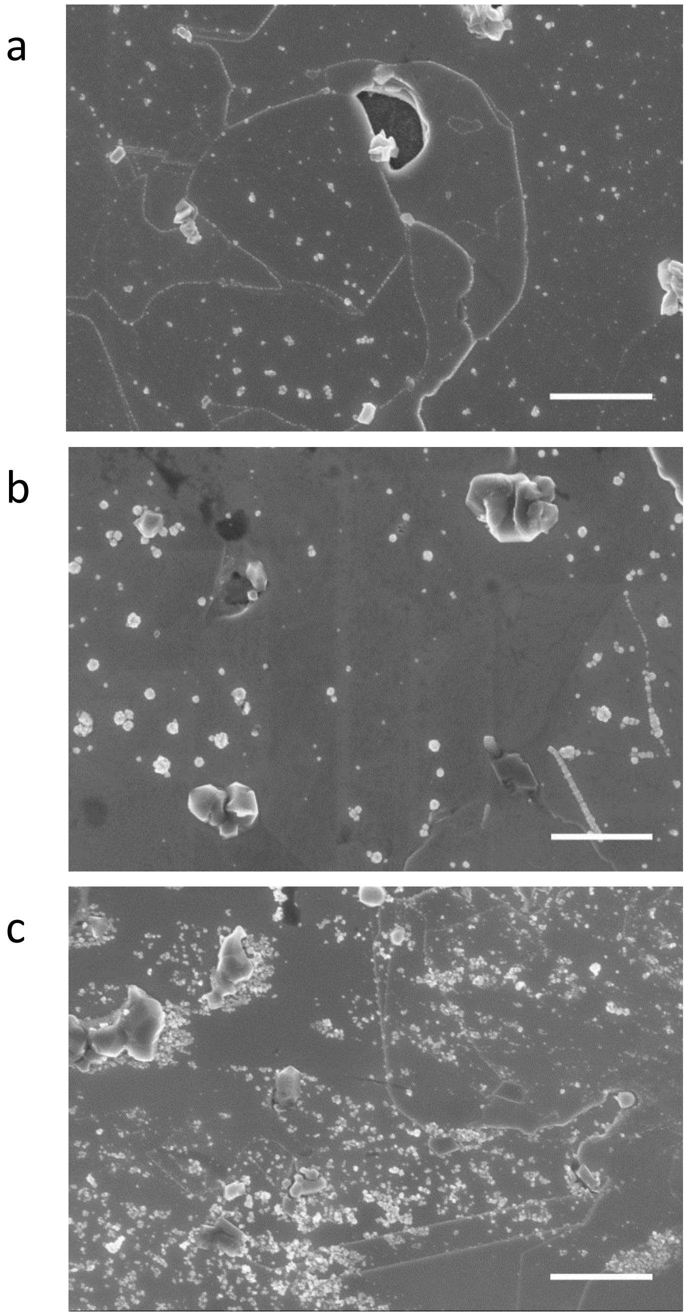

Figure S8. SEM images of a photochemically Pt-coated WSe_2 film at **(a)** 6 cycles (180 sec), **(b)** 9 cycles (270 sec) and **(c)** 12 cycles (360 sec) in top view, respectively. Scale bar is 1 µm. The shadowing of the WSe_2 film by Pt nanoislands with increasing deposition time.

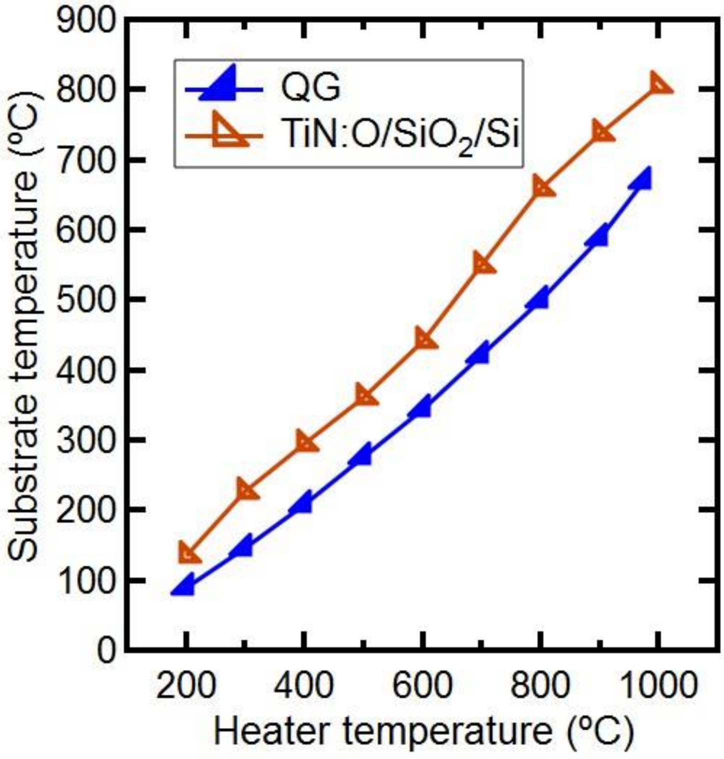

Figure S9. Calibration of the QG and TiN:O/SiO_2/Si substrates' temperatures with respect to the heater temperature.
